# Automated Amharic News Categorization Using Deep Learning Models

**DOI:** 10.1155/2021/3774607

**Published:** 2021-07-27

**Authors:** Demeke Endalie, Getamesay Haile

**Affiliations:** Faculty of Computing and Informatics, Jimma Institute of Technology, Jimma, Ethiopia

## Abstract

For decades, machine learning techniques have been used to process Amharic texts. The potential application of deep learning on Amharic document classification has not been exploited due to a lack of language resources. In this paper, we present a deep learning model for Amharic news document classification. The proposed model uses fastText to generate text vectors to represent semantic meaning of texts and solve the problem of traditional methods. The text vectors matrix is then fed into the embedding layer of a convolutional neural network (CNN), which automatically extracts features. We conduct experiments on a data set with six news categories, and our approach produced a classification accuracy of 93.79%. We compared our method to well-known machine learning algorithms such as support vector machine (SVM), multilayer perceptron (MLP), decision tree (DT), XGBoost (XGB), and random forest (RF) and achieved good results.

## 1. Introduction

Text categorization is the process of predicting the domain area of a given document [1]. With increasing in volume of text in today's computing world, text classification is highly required. Text classification can be performed either manually or automatically. Manual classification of documents requires a large workforce and wastes money. Furthermore, automatic document classification plays an important role in a variety of applications, including e-mail spam filtering, electronic document indexing, and online tendering. As a result, automatic text categorization is essential for dynamic management of information on Internet.

Amharic is a Semitic language that is also known as “Amarigna” and is one of Ethiopia's most widely spoken languages. It is spoken by up to 35 million Ethiopians, accounting for roughly one-third of the country's population [2]. The language's history can be traced back to the first millennium BC when refugees from southwestern Arabia crossed the Red Sea into modern-day Eritrea and mingled with the Cushitic people. The number of Amharic electronic documents is growing at the moment. Due to this, automatic Amharic text classification is highly required.

Text classification is well studied in many languages and, in particular, the English language [3]. Despite the fact that various works have been done to develop an Amharic document classifier, they all rely on machine learning models [4–6]. One of the most difficult problems in machine learning is reducing the dimensionality of a high-dimensional feature space. Dimensionality reduction has a direct impact on the model's overall performance. This problem can be solved by defining threshold values on the feature set. However, defining a threshold value for a machine learning model for dimensionality reduction, on the other hand, is complicated and time-consuming and may not yield consistent results for complex and massive data sets [7].

**Figure 1:**
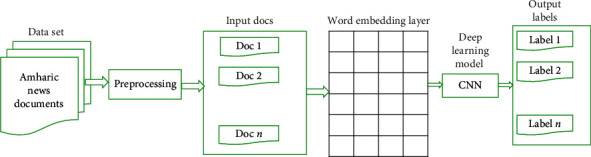
A general process of text classification using deep learning models.

CNN uses convolving filters in the convolutional layer to extract high-level features. It is extremely difficult to determine the number of filters and the appropriate size of a filter for a specific task. Larger filters consume more resources during training, whereas small filters may produce inaccurate results [8, 9]. CNN is capable of capturing contextual information for each feature.  Figure 1 depicts the overall process of text classification using CNN.

Since the past two decades, machine learning methods have been widely used for Amharic document classification, but the potential of deep learning has not been exploited yet. To the best of our knowledge, no deep learning models for long Amharic document classification have been applied. Deep learning models have drawn the attention of researchers from all fields of science due to their dependable and superior performance [10].

The aim of this study is to improve the classification accuracy of Amharic documents using deep learning and pretrained fastText. Our contributions are listed as follows:

1. To the best of our knowledge, it is the first study of document-level deep-learning-based Amharic text classification
2. We compared the deep learning model's performance to that of well-known machine learning models and found that the deep learning model outperforms

The rest of the paper is organized as follows. Related works are listed and explored in Section 2. The detail methodology of the proposed work is presented in Section 3. The experimental findings are defined in Section 4, we provide the results in Section 5, and the paper is concluded in Section 6.

## 2. Related Works

In recent years, many researchers have worked on deep learning-based document classification. Using a deep learning model for document classification improves classification accuracy, according to these recent studies. Even though, there is a lack of attempts on deep-learning-based text classification for Amharic language, we review existing works related to the proposed scheme mainly considering previous attempts made on text classification with Semitic language groups.

In the work of [11], authors investigated the performance of deep learning models by introducing new single label and multilabel data sets for Arabic text categorization. To improve the classification accuracy of Arabic text classification using deep learning models, they employ the word2vec embedding.

The authors in [12] proposed text classification based on CNN and word embedding for low-resource languages (Tigrinya). They used manually annotated data sets of 30,000 Tigrinya news texts from various sources, which are divided into six categories: sport, agriculture, politics, religion, education, and health. The authors construct CNNs using a variety of techniques, including continuous bag-of-words (CBOW), skip-gram, and CNNs with and without word2vec and fastText. fastText and word2vec pretrained vectors are compared in terms of their impact on text classification in their work. CNN with word2vec outperforms the other methods in terms of classification accuracy for Tigrinya news article classification, according to their experiment.

The works of [13] proposed a noble incremental learning strategy to solve the feature extraction problem in deep learning in text classification. Their model consists of four components: a student model, a reinforcement module, a teacher module, and a discriminator module. The learn# method significantly reduced the classifier's training time, reduced it by 80%. The author did not discuss the impact of their model on classifier performance in terms of accuracy, precision, recall, and F-measure.

In [14], the authors proposed an attention-based bi-directional long short-term memory (Bi-LSTM) +CNN hybrid model text classification. The proposed model capitalizes on the advantages of LSTM and CNN by including an additional attention mechanism. They tested their model using movie review data from the Internet Movie Database (IMDB), and the results showed that their proposed hybrid attention-based Bi-LSTM + CNN model outperforms MLP, CNN, and LSTM models in terms of classification accuracy, recall, and F1 scores.

The work of [15] studies the application of deep learning in text categorization. They combine it with textual characteristics and use the double bi-directional gated recurrent unit (GRU) + attention deep learning model to predict hotspots, and they achieved good results.

In reviewing the above papers, we noted that using deep learning model for text classification can enhance its performance. As a result, we proposed CNN + pretrained fastText that aimed for improving the performance of Amharic text classification.

## 3. Proposed Method

The components of the proposed document classification model for Amharic language are explained in this section. To evaluate and classify Amharic news documents, the proposed model includes four basic modules: data collection and preprocessing, data representation, classification module, and evaluation module. The proposed model of text classification is implemented according to the Algorithm 1.

**Figure 2:**
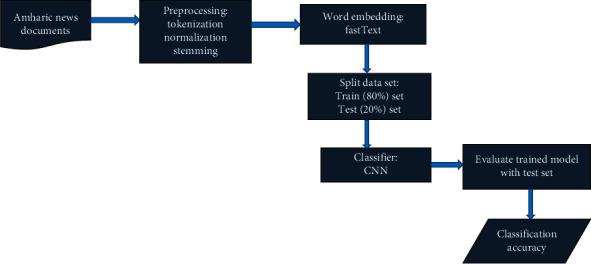
Architecture of the proposed model of Amharic text classifier.

The basic tasks or modules we used to predict the category label of raw Amharic document is described in Figure 2.

The description and function of each of the modules shown in Figure 2 is presented as follows.

### 3.1. Data Collection

We collected 3,600 Amharic news documents from 6 major news categories to train and test the designed deep learning model, since there is no publicly available Amharic document classification corpus. The six news categories are chosen based on the most commonly used category in previous studies. Each document file is saved in separate file name within the corresponding category's directory, that is, all documents in the data set are single labeled.

**Figure 3:**
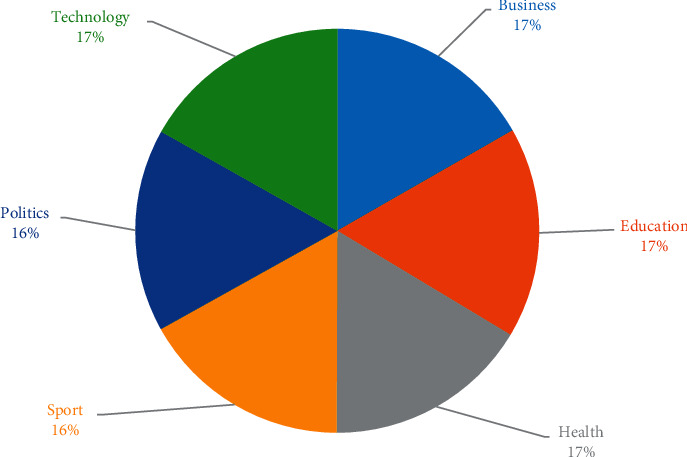
Data set composition.

The main goal of the proposed model is to predict the category of Amharic news document collected from different news publishing mass media such as Ethiopian News Agency (ENA), Fana Broadcasting Corporate (FBC), and Walta Information Center (WIC). ENA publishes news documents on a wide range of topics, such as sports, business, education, technology, health, and politics. The data sets for this analysis were collected from the FBC, WIC, and ENA websites. The six news categories used in this analysis are business, education, health, technology, politics, and sports.  Figure 3 shows the percentage of each news category in the data set as a pie chart.

### 3.2. Data Preprocessing

The process of preparing raw data for a machine learning or deep learning model is known as data preprocessing. Text cleaning is the first step of preprocessing. Before forwarding a raw data set to the learning model, it must be preprocessed. Since there may be noise and irrelevant data in a raw data set, it must be preprocessed before passing to the learning model, which increases the training time and degrades the model's efficiency. The following are some of the text cleaning techniques we used in this study.

### 3.3. Normalization

In the Amharic writing system, there are different characters with the same sound, and there is no written rule for using them in Amharic words. As a result, there is inconsistency in writing of terms. For example, the word power (“hayil”) can be written as ሀይል ፣ ሐይል ፤ ኀይል፤ ሃይል፤ሓይል፤ኃይል፤ኻይል. Characters of the same sound are replaced with the canonical form used in this study.  Table 1 shows normalization of Amharic characters having the same sound with different symbolic representation.

### 3.4. Stemming

The process of removing infixes (ላ፣ጃ፣ጫ), prefixes (በ፣ስለ፣እንደ፣ከ፣ወደ), and suffixes (ኦች፣ኦችም፣ን፣ም፣ንም) from words to produce the stem or root form is known as stemming [16]. In information processing, morphological variants of Amharic words have the same interpretation. HornMorpho 4, developed by Gasser [17], was used for stemming. HornMorpho generates morphemes from a given word, and we only use the root or stem form for the next text processing step. For example, it produced the lemma “ጨዋታ” for the word “ጨዋታዎች” (games), and we used only the term's lemma for the remaining tasks.

### 3.5. Word Embedding

Document classification involves the transformation of documents into feature vectors. This can be done via bag of words (BoW), word2vec, TF-IDF, and fastText. Word embedding is foundational to natural language processing and represents the words in a text in an R-dimensional vector space, thereby enabling the capture of semantics, semantic similarity between words, and syntactic information for words.

#### 3.5.1. Bag of Word

BoW counts the number of times a word appears in a document.  Table 2 shows the BoW representation of the sentence “the club is effective” across three documents: doc1, doc2, and doc3.

#### 3.5.2. Term Frequency-Inverse Document Frequency

The document's terms are not all equally important in distinguishing one document from the others. To determine the significance of terms in a classification, the TF-IDF method is used. This method does not rely on frequency, but rather on the TF-IDF score of each term or word throughout the document. The algorithm works similarly to bag of words, but the word count is replaced with the TF-IDF score of each term. TF-IDF score of a given term *t* in a document can be formulated as follows:

(1) $$\begin{array}{c} \text{TF}*\text{IDF}\left(t,d\right)={\text{TF}}_{t,d}*\;\log\;\;N/{\text{DF}}_{t}, \end{array}$$

where *N* denotes the total number of documents in the document, DF denotes document frequency, *t* denotes the term, and *d* denotes the document.

#### 3.5.3. word2vec

Word embedding via word2vec was proposed by Mikolov [18]. word2vec is a model that helps us represent the distributed representation of words in a corpus. In general, word2vec is an algorithm that accepts text as input and returns vectored representations of that text as output. word2vec starts with a set of random vector terms that scan the data set in a logical order while maintaining a background window around each word and its neighbors. The target word and its context are used by word2vec to decide how they act when they traverse the corpus [18].

The algorithm calculates the dot product between the target word and the background words and attempts to reduce the stochastic gradient descent (SGD) metric efficiency. Even when in a similar context, two words meet their relation, or spatial distance is enhanced.

#### 3.5.4. fastText

Facebook provides pretrained word vectors for 157 languages, including Amharic [19]. These models were trained with CBOW with position weights in dimension 300, character *n*-grams of length 5, a window of size 5, and 10 negatives. fastText is a Facebook-developed model for word representation and text classification that employs unsupervised approach to word representation. It is an extension of word2vec that views the representation of words from different directions. One of fastText's unique features is its ability to generate vectors for out-of-vocabulary words such as unknown words and tokens.

During word representation learning, fastText considers not only the word itself but also groups of characters from that word and subword information such as character unigrams, bigrams, and trigrams [20]. However, GloVe and word2vec fail to provide any vector representation for words that are not in the model dictionary [21]. As a result, in this study, fastText is used as a word representation model. We downloaded the fastText pretrained embedding model for Amharic language from the link https://fasttext.cc/docs/en/crawl-vectors.html and then used it to generate the vector of words found in our preprocessed data set. The pretrained word vectors were found in two formats: binary and text. We used the binary format for this study. The algorithm to get the vector of each word in the data set is shown in Algorithm 2.

**Figure 4:**
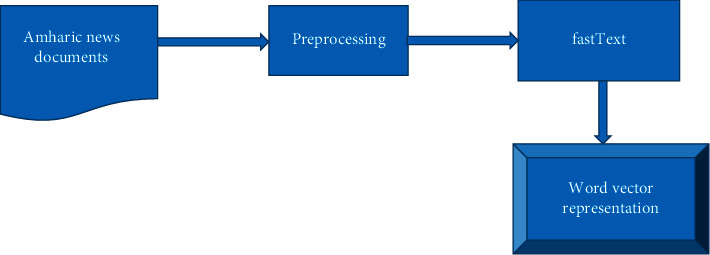
Word embedding model.

Then the generated matrix is used by the embedding layer of CNN. The modules used to generate word vectors for each term in the corpus are depicted in Figure 4.

### 3.6. Classification

**Figure 5:**
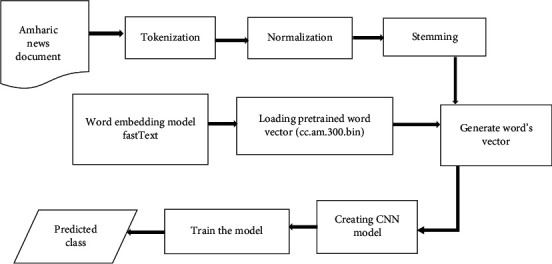
Block diagram of the proposed method.

With the increasing availability of Amharic documents on the internet, the proposed method uses convolution neural networks (CNNs) as a classifier method to classify certain documents. CNNs are advanced neural network models that are used to discover patterns and relationships between data items based on their relative positions [11]. CNNs can automatically learn effective text features representation from massive text using a 1D structure (word order) in the convolutional layer. It captures local relationships among the neighbor words in terms of context windows, and by using pooling layers, it extracts global features. CNN is a neural network made up of several convolutional and pooling layers. The block diagram of our proposed CNN-based text classification is shown in Figure 5.

The layers used in our models are described as follows.

#### 3.6.1. Embedding Layer

The words of a document are represented as word vectors by the embedding layer. Every word in the text document is converted into a fixed-size dense vector. To map input token sequences to word vectors, the embedding layer employs various embedding techniques. Word embedding generates word representations that can be fed into the convolution layer, which learns to identify text classes based on the input vector sequences.

#### 3.6.2. Convolutional Layer

A convolutional layer is made up of neurons that calculate the output in two steps: first calculate a weighted sum and then output using a nonlinear activation function on the sum. The convolution layer applies different-width filters to the news embedding matrix to extract distinct features as vectors corresponding to each filter and generates a feature map. The convolution word filter considers positions that are independent for every word, and filters at higher layers capture syntactic or semantic associations between phrases that are far apart in a text.

#### 3.6.3. Pooling Layer

The pooling layer shrinks or reduces the size of the feature map by merging or selecting a high-valued feature from neighboring features, allowing the higher-level layers to deal with more global values. We employed 1-max-pooling. The main advantage of incorporating such a max-pooling layer into the network is that it reduces the number of parameters or weights and controls overfitting.

#### 3.6.4. Fully Connected Layer

In this layer, each neuron has full connections with all of the neurons in the previous layer. The connection structure is the same as with layers in classic neural network models. Dropout regularization is applied to the fully connected layer to avoid overfitting and therefore improve the generalization performance. When the training is finished, the parameters and learned weights are saved into a file, which can later be loaded and used to predict classes of unlabeled news. The fully connected layer is a traditional feedforward neural network (FNN) hidden layer. It can be viewed as a subset of the convolutional layer with kernel size 1. This layer belongs to the trainable layer weights class and is used in the final stages of CNNs.

#### 3.6.5. Softmax Function

Finally, the vector representations in the fully connected layer are passed to the Softmax function, which returns the probability distribution over the labels as an output.

## 4. Experiment

In this section, we investigate CNN's performance with trained fastText for Amharic news documents. The results of the experiments are compared to current state-of-the-art machine learning models. All experiments are carried out in a Windows 10 environment on a machine equipped with a Core i7 processor and 16 GB of RAM. The number of epochs was used as a predictor in experiments. The details, settings, and evaluation methods used in the experiment are listed in the following.

### 4.1. Data Set

The total number of news documents used in our experiment is 3,600 (108,000 sentences) from the six news categories: business, education, health, politics, technology, and sport. We cleaned the data set and save in the.XLSX, where the label of the document was represented as integer value from 1 to 6. For business, education, health, politics, technology, and sport, we used 1, 2, 3, 4, 5, and 6, respectively. The total number of unique terms in our data set is 795,536.

### 4.2. CNN Training Parameters

We tuned hyperparameters of CNN models on data sets using a grid search to find the best value for each model parameter. Choosing the best model parameter equals getting the best model performance. We treat Amharic documents as an array of sentences separated by four points (:) for training purposes. We used a CNN-based approach to learn automatically and categorize those sentences into one of the six evaluation categories. CNNs require inputs to be a fixed size, whereas sentence lengths can vary greatly. As a result, we used a word length of 300 on average. For sentences with fewer than 300 words, the index is filled by appending a zero at the end until it reaches 299 index. The network parameters were regularized with a dropout rate of 0.5 and a training epoch typical value of 50. We divide the data set in an 80–20 ratio and evaluate the model in terms of accuracy, F-measure, recall, and precision.  Table 3 shows the CNN hyperparameters used in this study.

### 4.3. Performance Measure

We used the most widely used performance assessment metrics to assess classification performance: accuracy, precision, recall, and F-measure [22].

Accuracy is the most common metric for classifier efficiency and can be calculated as follows:

(2) $$\begin{array}{c} \text{accuracy}=\frac{\text{TP}+\text{TN}}{\text{TP}+\text{TN}+\text{FP}+\text{FN}}. \end{array}$$

Precision is used to determine the correctness of a classifier's result and can be determined as follows:

(3) $$\begin{array}{c} \text{precision}=\frac{\text{TP}}{\text{TP}+\text{FP}}. \end{array}$$

The completeness of the classifier results is measured by recall. The following equation is used to calculate it:

(4) $$\begin{array}{c} \text{recall}=\frac{\text{TP}}{\text{TP}+\text{FN}}. \end{array}$$

F-measure is the harmonic mean of precision and recall and can be calculated as follows:

(5) $$\begin{array}{c} F-\text{measure}=\frac{\text{TP}+\text{TN}}{\text{TP}+\text{TN}+\text{FP}+\text{FN}}, \end{array}$$

where TP denotes true positive, TN denotes true negative, FP denotes false positive, and FN denotes false negative.

## 5. Results and Discussion

The proposed CNN model trained by fastText is evaluated in terms of accuracy, precision, recall, and F-measure using six Amharic news categories.

The performance of the proposed CNN-based Amharic news document classification on six news categories is shown in Table 4. In the four evaluation matrices, the system produced nearly identical results. As a result, the number of false positives and false negatives is approximately equal. Furthermore, the model's performance is evaluated using unlabeled documents, and the results are summarized in Table 5.

Table 5 shows that the proposed model correctly predicts label of three news. In addition to this, we conduct experiment on the performance of the new model by varying number of epochs.

**Figure 6:**
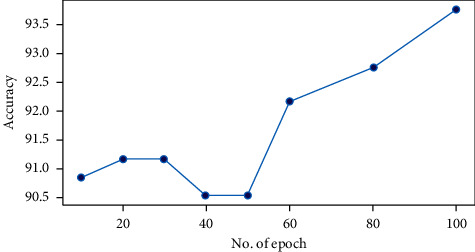
Evaluation of our model in terms of number of epochs.

As shown in Figure 6, we measured classification accuracy by varying the number of epochs. We used epoch 100 for the remaining experiments because it has the highest accuracy.

### 5.1. Comparison with Common Machine Learning Models

We compared our CNN model with the state-of-art machine learning models. The CNN model was evaluated and compared to the three most commonly used machine learning models, MLP, SVM, XGB, RF, and decision tree (DT) classifiers. We used models from the scikit-learn library from the “sklearn” package for the five machine learning models. To account for this variation in results, we ran it ten times without changing any parameters and took the average of the results. In addition, we run an experiment on SVM, MLP, and RF classifiers, varying the hyperparameter values, and the results are shown in Table 6.

From the above results, we took classifier parameters that resulted in better classification accuracy for SVM, MLP, and RF, namely, SVM (kernel = 'rbf'; *C* = 1.0; gamma = 1), MLP (hidden_layer_sizes = (200); max_iter = 1000), and n_estimators = 300 for the RF classifier for the comparison.  Table 7 shows a comparison of classification accuracy between the proposed mode and other machine learning classifiers.

In Table 7, the accuracy of the CNN-based method outperforms other methods, proving the effectiveness of this classification method. The following is an explanation of why performance should be improved in the CNN model: (1) using fastText to produce word vectors can get relevant features, and (2) processed text features through CNN can be better in representing high-level features of a document.

## 6. Conclusion

This paper focuses on a text categorization method based on CNN that does not require the extraction of text features in advance. CNN is used to extract higher-level features and improve network recognition. Simultaneously, prevent data from overfitting in the dropout method to improve the network's capacity. CNN's text classification performance is evaluated using various CNN parameters and epochs, and it is also compared to well-known machine learning classifiers. According to the experimental results, the proposed CNN-based classifier produced a better classification accuracy of 93.79%. As a result, the proposed model can be used in a variety of applications that require Amharic document classification, such as automatic document organization, topic extraction, and information retrieval. We aimed to examine additional categories and data sets in the future.

## Figures and Tables

**Algorithm 1:**
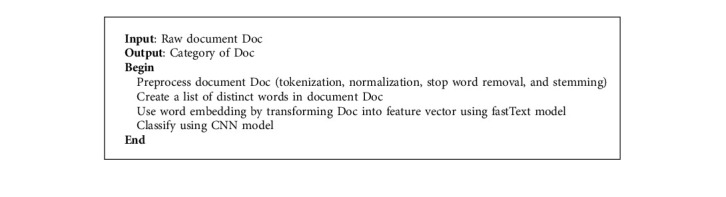
The framework for the proposed system.

**Algorithm 2:**
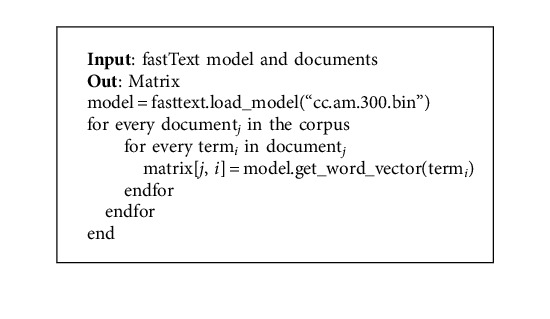
fastText implementation.

**Table 1:** List of consonants used by the study.

Canonical form	Characters to be replaced
hā(ሀ)	hā(ሃ፣ኃ፣ኀ፤ሐ፣ሓ)
se(ሰ)	se(ሠ)
ā(አ)	ā(ኣ፣0፣ዓ)
ts'e(ጸ)	ts'e(ፀ)
wu(ው)	wu(ዉ)
go(ጐ)	go(ጎ)

**Table 2:** Sample BoW representation.

Documents	The	Club	Is	Effective
doc1	10	3	4	4
doc2	5	1	7	2
doc3	12	0	2	6

**Table 3:** CNN model hyperparameters.

Hyperparameter	Value
Embedding dimension	300
Filter size	[3, 4, 5]
Number of filters	256
Batch size	64
Dropout	0.7
Activation	Softmax
Optimization	Adam

**Table 4:** Performance of model in terms of accuracy, precision, recall, and F-measure.

Evaluation metrics	Performance (%)
Accuracy	93.79
Precision	93.63
F-measure	93.67
Recall	93.76

**Table 5:** Model evaluation using unlabeled document.

No.	Sample documents translated from Amharic	Predicted label	Label by the expert	Status
1.	Every year, in Ethiopia, more people die of tuberculosis, according to the World Health Organization.	2 (Education)	3 (Health)	Failed

2.	Sports championship held in Oromia North Shoa and East Welega zones is over.	6 (Sport)	6 (Sport)	Accepted

3.	Prime Minister Dr. Abiy Ahmed spoke to the robot at his office.	5 (Technology)	5 (Technology)	Accepted

4.	Addis Ababa Ethio-Telecom has recently signed an agreement with eight companies to distribute Internet services.	5 (Technology)	5 (Technology)	Accepted

**Table 6:** Classification accuracy of SVM, MLP, and RF classifier with different hyperparameter values.

Classifier	Hyperparameters			Accuracy (%)
	Kernel	C	Gamma	
SVM	“Linear”	1.0	1	86.6
	“rbf”	1.0	1	88.88
	“rbf”	100	1	88,56
	“rbf”	10	1	88,51
	“Linear”	10	1	87.58

MLP	hidden_layer_sizes		max_iter	
	100		1000	87.57
	200		1000	88.48

RF	n_estimators			
	100			87.28
	200			88.23
	300			88.88

**Table 7:** Comparison of CNN model, FR, XGB, MLP, SVM, and DT.

Classifiers	Testing accuracy (%)
CNN	93.79
RF	88.88
XGB	87.58
SVM	88.88
MLP	88.48
DT	77.45

## Data Availability

This research work's data set and source code are publicly available on GitHub (https://github.com/demekeendalie/feature-selection).
